# High-risk behaviors and their association with awareness of HIV status among participants of a large-scale prevention intervention in Athens, Greece

**DOI:** 10.1186/s12889-020-8178-y

**Published:** 2020-01-28

**Authors:** Ioanna D. Pavlopoulou, Stavroula K. Dikalioti, Ilias Gountas, Vana Sypsa, Meni Malliori, Katerina Pantavou, Don Des Jarlais, Georgios K. Nikolopoulos, Angelos Hatzakis

**Affiliations:** 1 Pediatric Research Laboratory, National and Kapodistrian University of Athens, Faculty of Nursing, Athens, Greece; 2 Department of Hygiene, Epidemiology and Medical Statistics, National and Kapodistrian University of Athens, Medical School, Athens, Greece; 3 Hellenic Scientific Society for the Study of AIDS and Sexually Transmitted Diseases, Athens, Greece; 4 Psychiatric Department, National and Kapodistrian University of Athens, Medical School, Athens, Greece; 5 Medical School, University of Cyprus, P.O.Box 20537, Nicosia, Cyprus; 6 Icahn School of Medicine at Mount Sinai, New York, USA

**Keywords:** HIV, Awareness, Outbreak, PWID, High-risk behavior

## Abstract

**Background:**

Aristotle was a seek-test-treat intervention during an outbreak of human immunodeficiency virus (HIV) infection among people who inject drugs (PWID) in Athens, Greece that started in 2011. The aims of this analysis were: (1) to study changes of drug injection-related and sexual behaviors over the course of Aristotle; and (2) to compare the likelihood of risky behaviors among PWID who were aware and unaware of their HIV status.

**Methods:**

Aristotle (2012–2013) involved five successive respondent-driven sampling rounds of approximately 1400 PWID each; eligible PWID could participate in multiple rounds. Participants were interviewed using a questionnaire, were tested for HIV, and were classified as HIV-positive aware of their status (AHS), HIV-positive unaware of their status (UHS), and HIV-negative. Piecewise linear generalized estimating equation models were used to regress repeatedly measured binary outcomes (high-risk behaviors) against covariates.

**Results:**

Aristotle recruited 3320 PWID (84.5% males, median age 34.2 years). Overall, 7110 interviews and blood samples were collected. The proportion of HIV-positive first-time participants who were aware of their HIV infection increased from 21.8% in round A to 36.4% in the last round. The odds of dividing drugs at least half of the time in the past 12 months with a syringe someone else had already used fell from round A to B by 90% [Odds Ratio (OR) (95% Confidence Interval-CI): 0.10 (0.04, 0.23)] among AHS and by 63% among UHS [OR (95% CI): 0.37 (0.19, 0.72)]. This drop was significantly larger (*p* = 0.02) among AHS. There were also decreases in frequency of injection and in receptive syringe sharing in the past 12 months but they were not significantly different between AHS (66 and 47%, respectively) and UHS (63 and 33%, respectively). Condom use increased only among male AHS from round B to the last round [OR (95% CI): 1.24 (1.01, 1.52)].

**Conclusions:**

The prevalence of risky behaviors related to drug injection decreased in the context of Aristotle. Knowledge of HIV infection was associated with safer drug injection-related behaviors among PWID. This highlights the need for comprehensive interventions that scale-up HIV testing and help PWID become aware of their HIV status.

## Introduction

Despite significant achievements in terms of prevention and treatment, infection with human immunodeficiency virus (HIV) is still a global health challenge with around 38 million people living with HIV [1]. Many infections, especially outside Africa, are attributed to drug injection. In particular, 15.6 million people inject drugs (PWID) globally and of these, around 3 million are infected with HIV [2]. Moreover, HIV outbreaks have recently been observed among PWID in many European settings [3–7] and in one location in the United States (US) [8].

The HIV epidemic in Greece was concentrated mainly in men who have sex with men with sporadic HIV transmissions in PWID [9]. Harm reduction coverage had been constantly extremely low in the country, despite substantial evidence for prevalent risky behaviors among PWID [9]. For instance, in September 2011, the waiting list at facilities in Athens for medication-assisted treatment of opioid use disorder was 8 years. Before 2011, Needle/Syringe Programs (NSP) were distributing less than 20 syringes/needles per year per person who injected drugs [10]. After 2011, the epidemiological pattern changed dramatically. HIV diagnoses among PWID in Athens, the capital city of Greece, rocketed from less than 20 annually to more than 1000 in just 3 years (2011–2013), and drug injection became the most common route of transmission in 2012 [11]. The HIV outbreak occurred in the context of a financial distress that started in 2008 and soon turned into a serious economic, political, and social crisis [10].

In response to the rapidly evolving epidemic, there were efforts to increase HIV testing rates, to scale-up harm reduction measures, and to link PWID with antiretroviral treatment. The major element of the national response was Aristotle, a large-scale, seek-test-treat intervention [12, 13]. Aristotle used a respondent-driven sampling (RDS) approach to reach PWID, who constitute a hard-to-reach population, and ran multiple waves of HIV testing in 16 months in order to capture the largest possible proportion of infected PWID. The concept behind Aristotle was that by identifying HIV-undiagnosed PWID and helping them visit drug and HIV treatment facilities soon after diagnosis, HIV transmission rates will eventually decline. Aristotle collaborated closely and harmonically with non-governmental organizations and state agencies, especially the Greek Organization against Drugs (OKANA), which offered some of its offices in downtown Athens for Aristotle services delivery. Eventually, Aristotle managed to significantly decrease the HIV-undiagnosed fraction and HIV incidence among PWID in Athens, and the number of reported HIV cases has been rather stable in that group since 2014 [14].

It is important to understand if and how prevention approaches among PWID work. HIV testing and linkage to care help prevent HIV transmission by identifying undiagnosed HIV-infected people and helping them start potent antiretroviral treatment, which reduces viral load. HIV testing may also help in terms of prevention if people who receive an HIV diagnosis change behavior to protect their sexual or injecting partners from acquiring HIV. Awareness of HIV infection was correlated with lower prevalence of high-risk sexual behavior in a literature-based meta-analysis published in the early 2000s [15]. Previous research among PWID has produced conflicting results [16–23]. For instance, some studies showed that PWID who knew that they were HIV-infected were more likely to use condoms and less likely to give their used injecting equipment to other PWID [16, 19]. Other studies, however, found that risky behaviors were more prevalent among PWID who had received a diagnosis of HIV than among those who were unaware of their HIV infection or HIV negative [22]. There were also studies that found no association between HIV awareness and drug use behavior [23]. Notification of infection with another blood-borne pathogen, i.e., hepatitis C virus (HCV), has been associated with decreasing rates of syringe sharing in one study [24] and of drug injection in another [25].

Given the uncertainty concerning the association between HIV awareness and behavior among PWID and the opportunity to address it taking advantage of the longitudinal nature of Aristotle, the aims of this analysis were: (a) to study trends in high-risk drug injection-related and sexual behaviors among Aristotle participants over the course of the intervention; and (b) to compare the likelihood of high-risk drug injection-related and sexual behaviors between people who were knowledgeable of their HIV-positive status and people who did not know that they had been infected with HIV.

## Materials and methods

### Description of Aristotle

Aristotle was conceptualized, designed, and implemented by a research group in the Department of Hygiene, Epidemiology, and Medical Statistics at the Medical School of the National and Kapodistrian University of Athens, and ran between mid-2012 and end of 2013. The National Strategic Reference Framework 2007–2013, the European Social Fund, national resources, and the Hellenic Scientific Society for the Study of AIDS and Sexually Transmitted Diseases funded and supported this intervention. Aristotle focused on PWID and aimed to offer HIV testing, to increase the rate of HIV diagnosis, and to improve linkage to care.

In terms of design, Aristotle [11–13, 26] consisted of five successive enrollment rounds (A, B, C, D, E) using respondent-driven sampling. Each enrollment round lasted 10–12 weeks with between-rounds breaks of 1 to 4 weeks. Selected PWID acted as seeds to begin the chain referral process in each round (5–11 per round). Seeds received coupons and were asked to deliver them to three PWID from their social environments. The coupons had unique numbers linking each recruiter with his/her potential recruits.

PWID who visited the Aristotle site were eligible to participate in an RDS round if they: (i) held a valid RDS coupon (all but seeds); (ii) had been involved in drug injection without prescription in the past 12 months; (iii) were aged ≥18 years; (iv) were residents of Athens Metropolitan Area; and (v) came to the Aristotle site for the first time in the current testing round with the intention to participate (PWID could participate in multiple enrollment rounds but only once in each round). All participants gave written informed consent.

Experienced Aristotle staff interviewed the participants (computer-assisted personal interviews) based on the National HIV Behavioral Surveillance System (NHBS) questionnaire for PWID, appropriately adjusted to the Greek setting and with additional items that were of interest [27]. The questionnaire included items on demographics, injecting network size, accommodation status, history of incarceration, history of previous HIV testing, HIV status, sexual practices, and drug use history and practices.

Participants gave blood that was tested for HIV by a microparticle EIA anti-HIV-1/2 assay (AxSYM HIV-1/2 gO; Abbott Laboratories, Abbott Park, IL). Western blot (MP Diagnostics, Singapore) was used to confirm reactive results.

After the interview and the blood collection, participants received their primary monetary incentive, sterile syringes and injection paraphernalia, condoms, and leaflets. The monetary incentive included 5 Euros for participation in Aristotle (interview and blood collection); 3 Euros when the participant came back to receive his/her HIV-test results; and up to 9 Euros in total when his/her recruits were enrolled in the program. Those who tested HIV positive were referred to infectious disease clinics and to Opioid Maintenance Treatment (OMT) programs of the Greek Organization Against Drugs. Needle and syringes were also distributed by the Greek Organization Against Drugs and several non-governmental organizations [12, 13]. Participants also received three coupons to distribute them to other PWID. This recruitment chain resulted in the enrollment of approximately 1400 PWID in each round.

### Drug injection-related behaviors

Based on responses to relevant questionnaire items, high-risk drug injection-related behaviors were defined as follows: i) Injecting drugs at least once per day in the past 12 months; ii) Reporting receptive syringe sharing about half of the time, most of the time or all of the time the participant injected drugs in the past 12 months; iii) Reporting receptive syringe sharing the last time the participant injected drugs; iv) Practicing drug division with a syringe that someone else had already injected about half of the time, most of the time or all of the time the participant injected drugs in the past 12 months.

### Sexual behaviors

High-risk sexual behavior was operationalized as condomless sexual encounters (vaginal or anal sex) in the last year (participants answered “usually no” or “never” to the question about condom use). Males were asked whether they used condom with a female or male partner. Women PWID were asked about whether their male partner used condom when they had sex.

The exact wording of questions and responses for drug injection-related and sexual behaviors is given in the Additional file 1.

### HIV awareness

Participants were classified as aware of their HIV infection (AHS) if their self-reported HIV-positive status at the interview was in agreement with the positive result of their blood test. HIV-positive unaware (UHS) were those who did not know about their infection when they tested HIV positive. HIV-negative participants were those whose blood test was negative for HIV. Participants could be reclassified over the course of Aristotle if they had become aware of their HIV infection or had acquired HIV.

### Statistical analysis

Differences in demographic parameters and high-risk behaviors among the different groups were assessed using chi-squared tests and Kruskal–Wallis tests. A non-parametric test for trend was used to assess changes over time.

There were some parameters that dictated the selection of a certain advanced statistical method. First, some PWID participated in more than one enrollment rounds, which resulted in clustered data with multiple measurements on some of the participants at different times. Observations from the same participants may be correlated, while those from different subjects are assumed to be independent. Failure to account for correlation of within-subject measurements can result in invalid standard errors and erroneous findings. Second, drug injection-related and sexual behaviors were binary outcomes (high-risk versus less risky behavior or no risk) in these analyses measured repeatedly for some Aristotle participants. Awareness of HIV serostatus was also a time-dependent covariate. Multiple conventional logistic regression analyses of outcomes and covariates at each time-point (round) could inflate type I error rate. Third, high-risk behaviors might not change regularly over time, i.e., the probability of change may differ between segments of Aristotle rounds. In this case, a uniform regression function cannot be applied to the data. In order to accommodate the above-mentioned issues, piecewise linear generalized estimating equation models (GEE with logit link function) were selected to regress binary repeated outcomes (separate for each outcome) against covariates allowing for different slopes of change [24, 25, 28–30]. GEE are an extension of generalized linear models for longitudinal data and belong to the family of marginal models that take into account the averaged relationship in the population.

The choice of change point (or breakpoint) for the piecewise models was based on the graphical examination of the data and modelling procedures. Segmented GEE models were obtained by a two-piece definition of the regression function over two intervals of the domain of covariates (one segment from round A to round B and one segment from round B to E).

Given the potential correlation of measurements on the same subject, a working correlation (a hypothesized relationship between repeated observations on a participant) has to be defined a priori for the estimation of model parameters. These analyses assumed an unstructured correlation within each Aristotle participant.

All analyses were performed in Stata 11.1 [31].

## Results

### Sociodemographic characteristics, drug injection-related and sexual behaviors of all participants at their first visit (it could be in any sampling round) in Aristotle

Overall, 3320 PWID were enrolled in Aristotle across all 5 rounds. Of these, 53.8% participated only in one round, 20.5% in 2 rounds, 14.1% in 3 rounds, 11.3% in 4 rounds, and 7.9% in all rounds. Therefore, the total number of interviews was 7110. Sociodemographic characteristics and risk behaviors at first participation in Aristotle by gender are shown in Table 1. Participants were mostly males (2807, 84.5%) and from Greece; one third were homeless, currently or in the past 12 months; 64.1% said that they lacked health insurance; and almost half had history of imprisonment. The median age was 34.2 years.

**Table 1:** Baseline sociodemographic characteristics, and drug injection-related and sexual behaviors by gender among all participants at their first visit in Aristotle (2012-2013, N=3320 People Who Inject Drugs)

	Gender			
	Male	Female	Overall	*p*-value
	N (%)	N (%)	N (%)	
Origin				< 0.001
Other	479 (17.1)	48 (9.4)	527 (15.9)	
Greek	2327 (82.9)	465 (90.6)	2792 (84.1)	
Education, highest level completed				< 0.001
Up to Primary	811 (29.2)	96 (18.8)	907 (27.6)	
Middle/Secondary School	871 (31.4)	133 (26.0)	1004 (30.5)	
High school	799 (28.8)	173 (33.8)	972 (29.6)	
University	294 (10.6)	110 (21.5)	404 (12.3)	
Homeless				0.073
not homeless	1862 (66.5)	320 (62.4)	2182 (65.9)	
homeless in the last year, not now	294 (10.5)	70 (13.6)	364 (11.0)	
homeless now	642 (22.9)	123 (24.0)	765 (23.1)	
Ever having health insurance				0.021
No	1813 (64.9)	304 (59.6)	2117 (64.1)	
Yes	979 (35.1)	206 (40.4)	1185 (35.9)	
Ever participated in OMT				< 0.001
No	2110 (76.1)	312 (61.7)	2422 (73.9)	
Yes	661 (23.9)	194 (38.3)	855 (26.1)	
Ever been in prison				< 0.001
No	1382 (49.4)	311 (60.9)	1693 (51.2)	
Yes	1413 (50.6)	200 (39.1)	1613 (48.8)	
Main substance of use				< 0.001
Heroin	2318 (83.2)	381 (74.7)	2699 (81.9)	
Cocaine	361 (13.0)	101 (19.8)	462 (14.0)	
Buprenorphine	23 (0.8)	2 (0.4)	25 (0.8)	
Sisha/Methamph	16 (0.6)	1 (0.2)	17 (0.5)	
Speedball	67 (2.4)	25 (4.9)	92 (2.8)	
Frequency of injecting drugs in past 12 months				0.005
More than once a day	877 (31.3)	136 (26.5)	1013 (30.6)	
Once a day	218 (7.8)	29 (5.7)	247 (7.5)	
More than once a week	641 (22.9)	121 (23.6)	762 (23.0)	
More than once a month	416 (14.9)	73 (14.2)	489 (14.8)	
Less than once a month	647 (23.1)	154 (30.0)	801 (24.2)	
Receptive syringe sharing on last injection				0.131
No	2080 (79.8)	364 (76.8)	2444 (79.4)	
Yes	525 (20.2)	110 (23.2)	635 (20.6)	
Receptive syringe sharing in past 12 months				0.166
Never	1867 (67.1)	324 (63.7)	2191 (66.5)	
Rarely	691 (24.8)	147 (28.9)	838 (25.4)	
About half of the times	136 (4.9)	19 (3.7)	155 (4.7)	
Most of the times, Always	90 (3.2)	19 (3.7)	109 (3.3)	
Dividing drugs with a syringe someone else had used before about half of the times or more in the past 12 months				0.198
No	1774 (63.2)	309 (60.2)	2083 (62.8)	
Yes	1032 (36.8)	204 (39.8)	1236 (37.2)	
Frequency of condom use				< 0.001
Always/Usually yes	1251 (56.7)	165 (37.6)	1416 (53.5)	
Never/Usually no	957 (43.3)	274 (62.4)	1231 (46.5)	
	Median (IQR)	Median (IQR)	Median (IQR)	*p*-value
Median age in years	34.6 (30.3, 41.1)	32.7 (28.9, 38.5)	34.2 (30.1, 40.7)	< 0.001
Duration of injection drug use in years	13.0 (6.0, 19.0)	11.0 (6.0, 17.0)	12.0 (6.0, 18.0)	0.005

*IQR* interquartile range, *OMT* opioid maintenance treatment

The median duration of injection drug use was 12 years and the primary substance of use was heroin. More than one third of the participants reported at least daily injection of drugs with a median of 3 injections in a usual day; 20.6% reported receptive syringe sharing when they last injected; 8.0% reported receptive syringe sharing at least half the times they injected drugs in the past 12 months; and 37.2% said they divided drugs at least half of the times they injected drugs in the past 12 months with a syringe that someone else had used before.

More than half of the male participants (56.7%) reported that they consistently (always or usually yes) used condoms while 37.6% of female participants said so when they were asked about use of condoms by their male partners (Table 1).

### Sociodemographic characteristics, drug injection-related and sexual behaviors by awareness of HIV infection at first participation in Aristotle

At first participation in Aristotle, of 3320 PWID, 506 (15.2%) were positive for HIV. Of the HIV-positives, 127 (25%) were aware of their infection. Awareness of HIV infection at first participation was associated with female gender, Greek nationality, history of participation in OMT programs, and less frequent drug injection (less than once per day) in the past 12 months (Table 2). Moreover, HIV positive females who knew about their infection at their first visit to Aristotle were more likely to report that their male partners used condoms than HIV positive females who were not aware of their HIV status (*p* = 0.022). In males, condom use was not associated with awareness of HIV infection at their first visit to Aristotle (Table 2).

**Table 2:** Sociodemographic characteristics, drug injection-related and sexual behaviors by awareness of HIV infection at first participation (Aristotle, 2012–2013, *N* = 506 HIV-positive People Who Inject Drugs)

Variable	Awareness of HIV infection			
	Aware (*N* = 127)	Unaware (*N* = 379)	Overall	*p*-value^*^
	N (%)	N (%)	N (%)	
Gender				< 0.001
Male	87 (68.5)	332 (87.6)	419 (82.8)	
Female	40 (31.5)	47 (12.4)	87 (17.2)	
Median age in years (interquartile range)	34.1 (29.3, 38.5)	32.7 (28.5, 38.3)	33.1 (28.9, 38.3)	0.223
Origin				0.012
Other	16 (12.6)	87 (23.0)	103 (20.4)	
Greek	111 (87.4)	292 (77.0)	403 (79.6)	
Ever participated in OMT				< 0.001
No	61 (48.4)	306 (82.0)	367 (73.5)	
Yes	65 (51.6)	67 (18.0)	132 (26.5)	
Ever been in prison				0.057
No	40 (31.5)	155 (41.0)	195 (38.6)	
Yes	87 (68.5)	223 (59.0)	310 (61.4)	
Main substance of use				0.138
Heroin	85 (67.5)	287 (76.5)	372 (74.3)	
Cocaine	31 (24.6)	58 (15.5)	89 (17.8)	
Buprenorphine	0 (0.0)	2 (0.5)	2 (0.4)	
Sisha/Methamph	0 (0.0)	2 (0.5)	2 (0.4)	
Speedball	10 (7.9)	26 (6.9)	36 (7.2)	
Frequency of injecting drugs in past 12 months				0.035
More than once a day	55 (43.3)	211 (55.7)	266 (52.6)	
Once a day	11 (8.7)	24 (6.3)	35 (6.9)	
More than once a week	22 (17.3)	73 (19.3)	95 (18.8)	
More than once a month	15 (11.8)	31 (8.2)	46 (9.1)	
Less than once a month	24 (18.9)	40 (10.6)	64 (12.6)	
Receptive syringe sharing on last injection				0.889
No	83 (74.8)	249 (74.1)	332 (74.3)	
Yes	28 (25.2)	87 (25.9)	115 (25.7)	
Receptive syringe sharing in past 12 months				0.741
Never	66 (52.4)	190 (50.7)	256 (51.1)	
Rarely	40 (31.7)	131 (34.9)	171 (34.1)	
About half the times	10 (7.9)	33 (8.8)	43 (8.6)	
Most of the times, Always	10 (7.9)	21 (5.6)	31 (6.2)	
Dividing drugs with a syringe someone else had used before about half of the times or more in the past 12 months				0.112
No	54 (42.5)	192 (50.7)	246 (48.6)	
Yes	73 (57.5)	187 (49.3)	260 (51.4)	
Condom use for males				0.719
Never/Usually no	32 (28.8)	57 (30.8)	89 (30.1)	
Always/Usually yes	79 (71.2)	128 (69.2)	207 (69.9)	
Condom use for females				0.022
Never/Usually no	10 (19.6)	13 (43.3)	23 (28.4)	
Always/Usually yes	41 (80.4)	17 (56.7)	58 (71.6)	

*OMT* opioid maintenance treatment

^*^for the comparison between participants who were aware and unaware of their infection

### Trends in drug injection-related behaviors

**Fig. 1:**
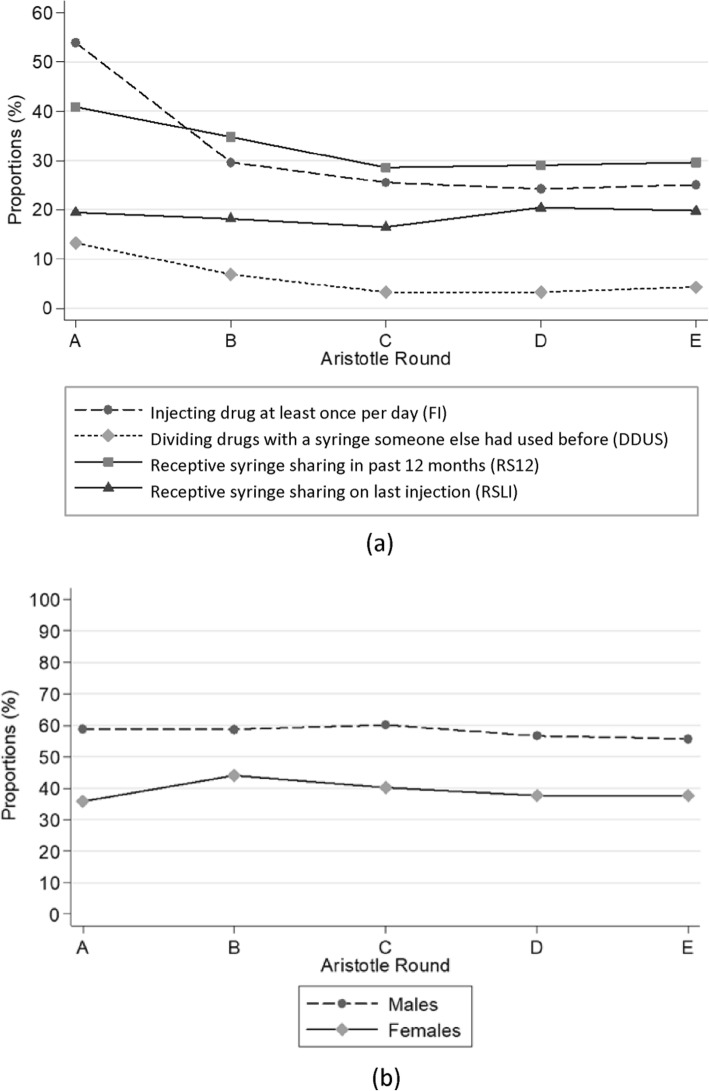
Prevalence of (**a**) drug injection-related behaviors and (**b**) condom use over the five rounds of Aristotle (round A to E)

Figure 1a shows changes in high-risk injecting behaviors across Aristotle rounds. The reported prevalence of high-risk drug injection-related behaviors including injecting at least once per day in the last 12 months, dividing drugs at least half of the times the participant injected drugs in the past 12 months with a syringe someone else had used before, and receptive syringe sharing at least half of the times the participant injected drugs in the past 12 months, declined significantly (p for trend < 0.001). There was no change, however, in the proportion of PWID who reported that they had shared a syringe the last time they injected.

### Trends in sexual behaviors

Males reported use of condoms more frequently than females throughout the course of Aristotle. The reported prevalence among males remained, more or less, the same in all rounds. In females, there was a small increase from round A to B but condom use gradually declined from the second to the last round. There was not however any statistically significant change in condom use among both males and females across the Aristotle rounds (Fig. 1b).

### Trends in prevalence of HIV and in the proportion of HIV-infected people who were aware of their infection among first-time participants in each round

**Fig. 2:**
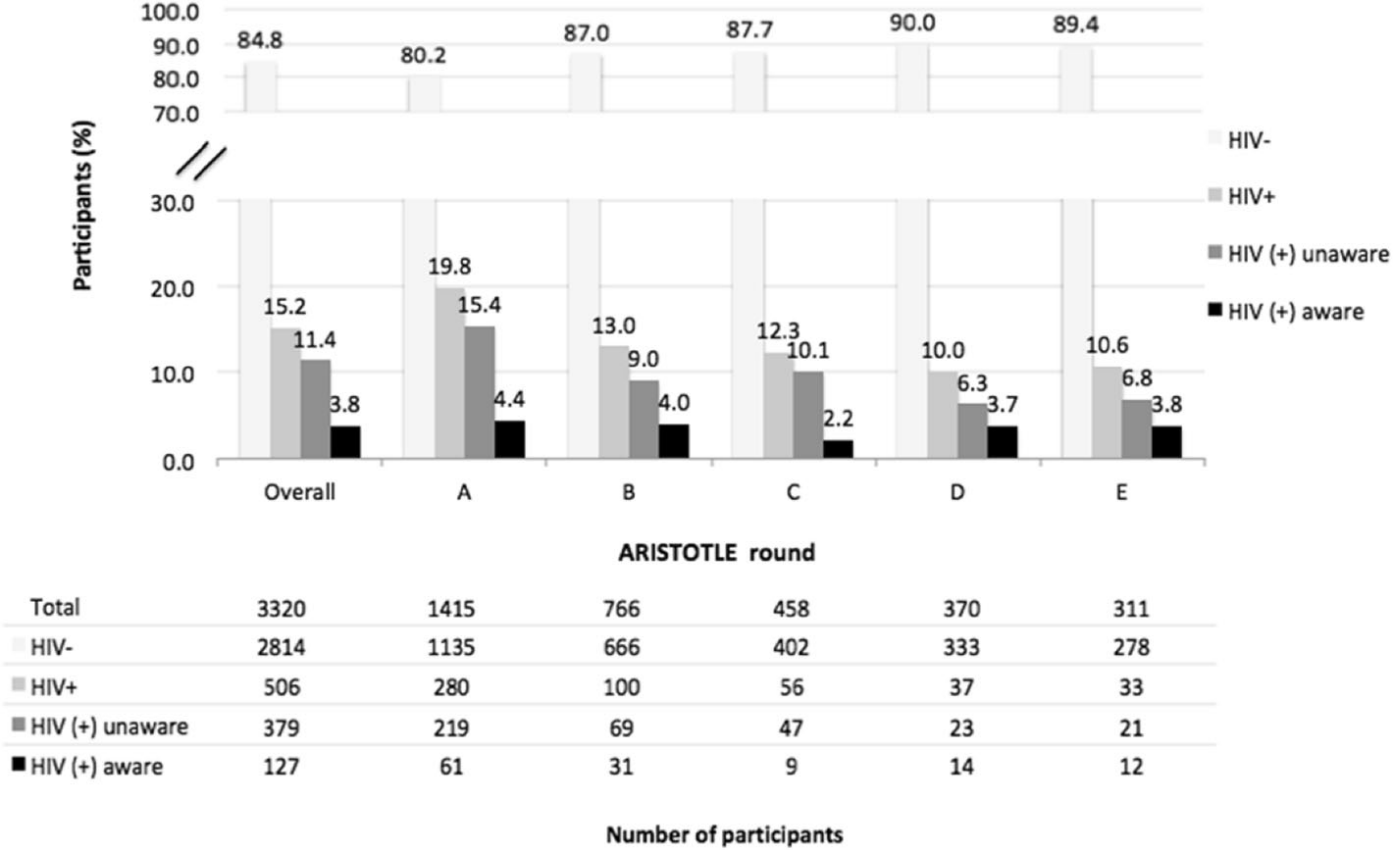
HIV status and awareness of HIV infection among participants in Aristotle at their first visit (overall and by round)

More than half of all HIV-positives (280/506; 55.3%) in Aristotle were found in the first enrollment round (Fig. 2). The prevalence of HIV among first-time participants decreased across rounds (19.8% in round A to 10.6% in the last round).

The proportion of HIV-infected participants who knew about their infection at their first visit in Aristotle increased from 21.8% (61/280) in round A, to 31% (31/100) in round B and to 36.4% (12/33) in the last round of the program (Fig. 2).

### Awareness of HIV serostatus and drug injection-related behaviors

**Fig. 3:**
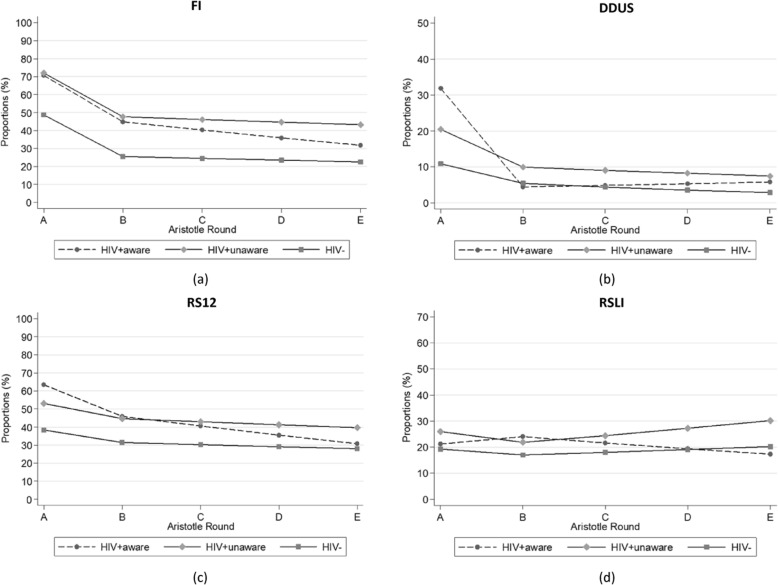
Trends in the reported prevalence of (**a**) injecting drugs at least once per day (Frequency of Injection – FI), (**b**) dividing drugs at least half of the times the participant injected drugs in the past 12 months with a syringe someone else had used before (DDUS), (**c**) receptive syringe sharing at least half of the times the participant injected drugs in the past 12 months (RS12), and (**d**) receptive syringe sharing on last injection (RSLI), by HIV status and awareness of HIV infection over the five rounds of Aristotle (A to E)

The prevalence of drug injection-related behaviors among AHS, including injecting drug use at least once per day in the past 12 months, receptive syringe sharing at least half of the times the participant injected drugs in the past 12 months, and dividing drugs at least half of the times the participant injected drugs in the past 12 months with a syringe someone else had used before, decreased from round A to round Ε (Fig. 3a, b, c, d).

Multivariable analyses using GEE models were adjusted for gender, age, origin, homelessness, education, history of imprisonment, and history of OMT. The odds of injecting drugs at least once per day in the past 12 months among AHS decreased by 66% [Odds Ratio (OR); 95% Confidence Interval (CI): 0.34 (0.19, 0.61)] from round A to B (Table 3). Similarly, among UHS, the odds of injecting drugs at least once per day in the past 12 months were 63% less in round B than in round A [OR (95% CI): 0.37 (0.25, 0.57]. Between rounds B and E, the odds of injecting drugs at least once per day in the past 12 months were slightly reduced by 16% [OR (95% CI): 0.84 (0.74, 0.96)] and by 6% [OR (95% CI): 0.94 (0.76, 1.18)] among AHS and UHS, respectively.

**Table 3:** Binary dependent variables measured repeatedly over the course of Aristotle (past 12 months: frequency of injecting drugs, receptive syringe sharing, dividing drugs with a syringe someone else had already used and condom use) regressed against covariates (Aristotle round and awareness of HIV status). Results from multivariable piecewise Generalized Estimating Equation analyses (Aristotle, 2012–2013, participants = 3320, observations = 7110, adjustment for gender, age, origin, homelessness, education, history of imprisonment, and history of Opioid Maintenance Treatment)

Awareness of HIV status	Injecting drugs at least once per day versus less than once per day	Dividing drugs at least half of the time the participant injected drugs with a syringe someone else had used before versus less than half of the time	Receptive syringe sharing at least half of the time the participant injected drugs versus receptive sharing less than half of times	Condom use “always” or “usually yes” versus “usually no” or “never”	
				Females	Males
Round B vs. Round A	OR (95% CI)				
HIV (+) aware (AHS)	**0.34 (0.19, 0.61)**	**0.10 (0.04, 0.23)**	**0.53 (0.31, 0.91)**	0.92 (0.25, 3.35)	1.65 (0.78, 3.50)
HIV (+) unaware (UHS)	**0.37 (0.25, 0.57)**	**0.37 (0.19, 0.72)**	**0.67 (0.45, 0.99)**	0.62 (0.22, 1.79)	1.35 (0.79, 2.29)
HIV (−)	**0.37 (0.32, 0.42)**	**0.48 (0.36, 0.64)**	**0.75 (0.65, 0.86)**	1.40 (0.95, 2.05)	0.99 (0.85, 1.17)
Round B vs. Round E					
HIV (+) aware (AHS)	**0.84 (0.74, 0.96)**	1.10 (0.81, 1.50)	**0.81 (0.71, 0.93)**	0.86 (0.64, 1.17)	**1.24 (1.01, 1.52)**
HIV (+) unaware (UHS)	0.94 (0.76, 1.18)	0.96 (0.64, 1.44)	0.96 (0.77, 1.20)	0.88 (0.50, 1.55)	1.21 (0.88, 1.67)
HIV (−)	0.95 (0.90, 1.01)	**0.80 (0.70, 0.91)**	0.96 (0.91, 1.01)	0.96 (0.85, 1.08)	**0.94 (0.89, 0.99)**

Statistically significant ORs are in bold

The odds of dividing drugs about at least half of the times the participant injected drugs in the past 12 months with a syringe someone else had used before (past 12 months) fell from round A to B by 90% [OR (95% CI): 0.10 (0.04, 0.23)] among AHS and by 63% among UHS [OR (95% CI): 0.37 (0.19, 0.72)]. The decline was significantly less (*p* = 0.02) among UHS than among AHS. There were not any significant changes in the odds of dividing drugs at least half of the times the participant injected drugs in the past 12 months with a syringe someone else had used before (past 12 months) from round B to E in either group.

Comparing round A to B, the odds of receptive syringe sharing at least half of the times the participant injected drugs in the past 12 months decreased by 47% in the round B among AHS [OR (95% CI): 0.53 (0.31, 0.91)] and by 33% among UHS [OR (95% CI): 0.67 (0.45, 0.99)]. Between rounds B and E, the odds of receptive syringe sharing at least half of the times the participant injected drugs in the past 12 months were reduced by 19% [OR (95% CI): 0.81 (0.71, 0.93)] among AHS and by 4% [OR (95% CI): 0.96 (0.77, 1.20)] among UHS.

HIV negatives experienced smaller decreases in the odds of high-risk drug injection-related behaviors from round A to B than AHS or UHS.

### Awareness of HIV serostatus and sexual behaviors

Reported condom use did not significantly change in both males and females between rounds A and B. From round B to E, condom use among AHS males increased significantly by 24% [OR (95% CI): 1.24 (1.01, 1.52)]. A non-significant increase in condom use (21%) was observed among UHS males [OR (95% CI): 1.21 (0.88, 1.67)]. However, this difference in changes of condom use between AHS and UHS did not reach statistical significance. Interestingly, between rounds B and E, there was a slight 6% decrease in condom use among male HIV-negative participants [OR (95% CI): 0.94 (0.89, 0.99)]. There were no changes in condom use from round B to E among AHS [OR (95% CI): 0.86 (0.64, 1.17)], UHS [OR (95% CI): 0.88 (0.50, 1.55)], and HIV-negatives [OR (95% CI): 0.96 (0.85, 1.08)] in the female population of Aristotle.

## Discussion

In response to a big outbreak among PWID in Athens, Greece, Aristotle, an RDS approach consisted of five successive rounds of HIV testing and subsequent linkage to care, was implemented. High-risk drug injection-related behaviors decreased over time, markedly from the first to the second enrollment round and slightly thereafter. Improvements were larger among HIV-positive participants who knew about their infection, especially regarding the practice of dividing drugs with a syringe someone else had used before. HIV-infected males also reported higher levels of condom use in the period between the second and the last round.

Only 25% of all HIV-positives identified in Aristotle were aware of their serostatus at their first visit to the program. This is rather striking but could be attributed to the fact that the outbreak in Athens was recent. This finding is in agreement with previous studies, which found that HIV-infected PWID remain unaware of their serological status for a certain time [20, 21, 32–38]. PWID might not seek HIV testing for a variety of reasons including stigma and discrimination [39–44], fear of finding out that they are infected, and their perception of being at low risk for HIV infection [38, 45]. Moreover, reduced access to HIV testing services or affordable antiretroviral treatment due to insufficient and incorrect knowledge, long waiting time, and lack of family support [46] may be another barrier for PWID to learning their serological status.

HIV-infected participants in Aristotle, who knew about their infection, were less likely over the course of the program than the HIV-infected participants who were unaware of their status to report high-risk injection-related behaviors, including daily drug injection, receptive syringe sharing, and especially dividing drugs with a used syringe. The role of awareness of HIV infection in behavior change among PWID remains unclear. Some research groups have reported that knowledge of HIV infection is associated with less high-risk injecting behaviors [16, 21, 47, 48]. However, other researchers have reported that the prevalence of high-risk behaviors was higher among AHS PWID than among UHS [20, 22] or that injection-related behavior is unrelated to the awareness of HIV serostatus [23]. The likely effect of HIV-awareness on behavior change in Aristotle could be explained by the fact that around half of its enrollees participated in several rounds, and were thus exposed to the multiplying effect of repeated counseling and referrals to OMT and infectious disease clinics. This longitudinal effect of Aristotle might partly also explain the smaller changes towards safer behaviors that were also observed among the HIV negative participants. In fact, the adoption of safer behaviors by all Aristotle participants are reflected to some degree in the sharp reduction of HIV incidence over the course of the program [14, 26].

Some studies have reported that HIV diagnosis and counseling increase the odds of condom use in HIV-serodiscordant couples [49], among males who were aware of their HIV+ status compared to those who were unaware of their HIV+ status [18] or among HIV positive females who are aware of their infection, as opposed to HIV-negatives [50]. In our study, although HIV-positive females who were aware of their serostatus were more likely to report condom use by their male partners at their first visit, their sexual behavior did not change throughout Aristotle. This could be explained perhaps by the fact that PWID who were informed of their infection and initiated antiretroviral treatment developed a perception of safety due to treatment receipt and thereby increased condomless sex [51, 52] despite counseling. Generally, condom use is remarkably low in stable relationships as opposed in commercial or casual sexual encounters, where is typically high [53, 54].

Reported condom use among males in Aristotle was not associated with awareness of HIV infection at their first visit to the program. However, there was a statistically significant increase in reported condom use among HIV-positive males between rounds B and E. This increase did not significantly differ between those who were aware and unaware of their infection. Previous research has shown that recently HIV-infected men who have sex with men reduce high-risk sexual behavior soon after their diagnosis as we also found for male PWID in Aristotle [55, 56]. However, a behavioral rebound towards riskier sexual behaviors was noted around 9 months after HIV diagnosis [56], which highlights the importance of intense counseling to HIV-positives during the first months following the diagnosis.

This analysis has a couple of limitations: a) Behavior-related data were self-reported, which raises concerns about potential social desirability bias. Given that illicit drug use and HIV-infection stigmatize people, PWID could under-report high-risk behaviors in order to be more socially acceptable during face-to-face interviews [15, 57]. Previous studies have shown, however, that self-reports are adequately valid for this type of research [58, 59]. In terms of the effect of the type of interview, researchers studying risk behaviors, including receptive syringe sharing among PWID, have not found differences between responses by audio-computer assisted self-interview (ACASI) technology and face-to-face interviews [15]. Other researchers, however, have observed over-reporting of socially accepted behaviors in face to face interviews [19, 57, 60]; b) The effect of awareness of HIV status on high-risk behaviors was observed in the context of a large-scale combination prevention program. For less intensive programs without integrated services, the generalizability of these findings is unknown; c) Aristotle was not a randomized intervention, which makes difficult to determine to what degree was causally related with outcomes such as incidence reduction or behavior change. However, the trends in drug injection-related behaviors among the participants were assessed in an unbiased way and the conclusions could be considered as valid. Moreover, a randomized intervention during an outbreak would be unethical.

Aristotle was unique as an RDS intervention in terms of size and coverage [12]. In a relatively short period of time (16 months) and in the context of a large HIV outbreak and severe financial obstacles, it identified 88% of the people who injected drugs in downtown Athens, tested them for HIV, and helped them access services [12]. In addition, Aristotle managed to get multiple estimates of both HIV incidence and prevalence over time [14], to inform molecular analyses of HIV transmission [61], and to capture changing behavioral patterns that put PWID at risk for HIV infection. At the same time, Aristotle collaborated nicely with drug treatment-related and hospital facilities, and non-governmental organizations, and established itself in the PWID community as a useful and safe intervention [12]. The success of Aristotle serves as an epidemiological and public health paradigm of effectiveness, efficiency, and collaboration that can inform and enrich future interventions. This is of particular importance given the multiple outbreaks that were observed since 2011 around the world, even in settings with high coverage of harm reduction services [62]. We believe that Aristotle-type approaches of much smaller size and coverage should be an essential component of routine public health practice but should also be able to rapidly expand as a response to emergencies.

## Conclusions

This analysis of Aristotle data showed that the prevalence of high-risk drug injection-related behaviors decreases during this type of interventions and PWID aware of their HIV-positivity are less likely over time to report high-risk drug injection-related behaviors. These improvements in behaviors may partially explain the observed great drop in HIV incidence among PWID over the course of Aristotle. Therefore, knowledge of HIV-infection among PWID seems to be important, which makes imperative to implement programs that scale-up HIV testing and help people become aware of their status.

## Supplementary information

**Additional file 1.** Exact wording of questions and responses for drug injection-related and sexual behaviors.

## Data Availability

The datasets generated and/or analysed during the current study are not publicly available because they include personal identifiers but anonymized datasets could be available from the corresponding author on reasonable request.

## Abbreviations

AHS: HIV-positive participants aware of their infection
CI: 95% Confidence Interval
DDUS: Dividing drugs with a syringe someone else had used before
FI: Frequency of Injection
GEE: Generalized estimating eq.
HCV: Hepatitis C Virus
HIV: Human Immunodeficiency Virus
NHBS: National HIV Behavioral Surveillance System
OMT: Opioid Maintenance Treatment
OR: Odds Ratio
PWID: People Who Inject Drugs
RDS: Respondent Driven Sampling
RS12: Receptive syringe sharing in past 12 months
RSLI: Receptive syringe sharing on last injection
UHS: HIV-positive participants unaware of their infection
