# Supplementary material for: High-risk behaviors and their association with awareness of HIV status among participants of a large-scale prevention intervention in Athens, Greece
Source: BMC Public Health. 2020 Jan 28;20:105. doi: 10.1186/s12889-020-8178-y (PMC6986033; doi:10.1186/s12889-020-8178-y)
Supplement: Supplementary file 1 — Additional file 1. Exact wording of questions and responses for drug injection-related and sexual behaviors. [file 12889_2020_8178_MOESM1_ESM.doc]

**Additional file 1**

**A) Variable on frequency of injection (FI)**

In the past 12 months, on average, how often did you inject?

1-More than once a day

2-Once a day

3-More than once a week

4-Once a week

5-More than once a month

6-Once a month

7-Less than once a month

99-Refused/Don't know

The answers were collapsed into high-risk behavior - 1 (more than once per day, once per day) and less risky - 0 (less than once per day)

**B) Variable on receptive syringe sharing in the past 12 months (RS12)**

In the past 12 months, how often did you use syringes that someone else had already injected with?

0-Never

1-Rarely

2-About half the time

3-Most of the time

4-Always

9-Refused/Don't know

The answers were collapsed in high-risk 1 (about half the time, most of the time, always) and less risky - 0 (never, rarely)

**C) Variable on receptive syringe sharing on last injection (RSLI)**

ID-16. The last time you injected with someone, did you use a syringe after someone else had already injected with it?

0-No

1-Yes

9-Refused/Don't know

**D) Variable on having divided drugs with an already used syringe (DDUS)**

In the past 12 months when you injected, how often did you use drugs that had been divided with a syringe that someone else had already injected with?

0-Never

1-Rarely

2-About half the time

3-Most of the time

4-Always

9-Refused/Don't know

The answers were collapsed into high-risk - 1 (about half the time, most of the time, always) and less risky - 0 (never, rarely)

**E) Variable on use of condoms**

With the partner/partners you had during the past year, did you have vaginal or anal sex using a condom?

0-Always

1-Usually yes

2-Usually no

3-Never

The answers were collapsed into high-risk - 1 (never use a condom or usually no use of a condom) and less risky - 0 (always use a condom or usually use a condom)
